# Protection of Alcohol Dehydrogenase against Freeze–Thaw Stress by Ice-Binding Proteins Is Proportional to Their Ice Recrystallization Inhibition Property

**DOI:** 10.3390/md18120638

**Published:** 2020-12-13

**Authors:** Young Hoon Lee, Kitae Kim, Jun Hyuck Lee, Hak Jun Kim

**Affiliations:** 1 Department of Chemistry, Pukyong National University, Busan 48513, Korea; Leeyh@pukyong.ac.kr; 2 Research Unit of Cryogenic Novel Material, Korea Polar Research Institute, Incheon 21990, Korea; ktkim@kopri.re.kr (K.K.); junhyucklee@kopri.re.kr (J.H.L.); 3 Department of Polar Sciences, University of Science and Technology, Incheon 21990, Korea

**Keywords:** ice-binding protein, ice recrystallization inhibition, alcohol dehydrogenase, antifreeze protein, freezing damage

## Abstract

Ice-binding proteins (IBPs) have ice recrystallization inhibition (IRI) activity. IRI property has been extensively utilized for the cryopreservation of different types of cells and tissues. Recent reports demonstrated that IRI can also play a significant role in protecting proteins from freezing damage during freeze–thaw cycles. In this study, we hypothesized that the protective capability of IBPs on proteins against freeze–thaw damage is proportional to their IRI activity. Hence we used two IBPs: one with higher IRI activity (LeIBP) and the other with lower activity (FfIBP). Yeast alcohol dehydrogenase (ADH) was used as a freeze-labile model protein. IBPs and ADH were mixed, frozen at −20 °C, and thawed repeatedly. The structure of ADH was assessed using fluorescence emission spectra probed by 1-anilinonaphthalene-8-sulfonate over the repeated freeze–thaw cycles. The activity was monitored at 340 nm spectrophotometrically. Fluorescence data and activity clearly indicated that ADH without IBP was freeze-labile. However, ADH maintained about 70% residual activity after five repeated cycles at a minimal concentration of 0.1 mg mL^-1^ of high IRI-active LeIBP, but only 50% activity at 4 mg mL^−1^ of low active FfIBP. These results showed that the protection of proteins from freeze–thaw stress by IBPs is proportional to their IRI activity.

## 1. Introduction

Ice-binding proteins (IBPs), including antifreeze proteins (AFPs), have a binding affinity for ice [1]. IBPs have been found in many organisms living in the regions where they undergo freezing and thawing [2]. The presence of IBPs seems to be inevitable for the survival of these organisms under subfreezing conditions [3,4,5,6]. Even at low concentration, IBPs are known to inhibit the ice recrystallization (IR) [7]. IR is a process of forming larger ice grains at the expense of smaller ones [8], and a fatal but frequent event that many organisms probably experience in the cold environment. The IR inhibition (IRI) of IBPs appears to offer protection to the cold-tolerant organisms from fatal freezing injury [7,9]. This IRI property of IBPs has been extensively utilized for the cryopreservation of different types of cells and tissues (reviewed by [2]). Compared to the thermal hysteresis (TH) activity of IBPs, IRI activity has been investigated for biotechnological application. Very recently Rodriguez et al. [10] showed that IRI activity of AFP can even protect a protein molecule from freezing stress. They used lactate dehydrogenase (LDH) as a freeze-labile model protein and *Dendroides canadensis* AFP (DcAFP) as a cryoprotective agent. DcAFP is a hyperactive AFP with IRI activity at concentrations as low as 20 μg mL^−1^ [11,12]. Recombinant DcAFP successfully protected LDH from repeated freeze–thaw cycles at the concentration of 25 μg mL^−1^. In addition, Mitchell et al. [13] also showed that polymers with IRI activity protected proteins from freezing damage. These results suggest the possible use of IBPs for the cryopreservation of many therapeutic proteins [14,15,16,17].

To further elucidate the protective role of IBPs on proteins, based on their IRI activity, in this study we used two IBPs: one from *Glaciozyma* sp., an Arctic yeast (LeIBP) [18], and the other from *Flavobacterium frigoris*, an Antarctic sea ice bacterium (FfIBP) [19]. The two IBPs have almost similar β-helical structure, sharing 56% sequence identity between them [18,19]. However, they have significant differences in activity: In TH, LeIBP is moderately active but FfIBP hyperactive, while in IRI LeIBP is active at concentrations as low as 1 μg mL^−1^, but FfIBP at only as low as 28 μg mL^−1^ [8,16,17,18]. In cryopreservation or protection from cryodamage, IRI activity seems to play a critical role [20,21,22,23,24]. As a freeze-labile model protein, we used alcohol dehydrogenase (ADH) from baker’s yeast. ADH is a tetrameric metalloenzyme consisting of identical or similar subunits with two zinc atoms per subunit [25]. Since ADHs are a valuable biocatalyst in the asymmetric synthesis of chiral compounds [26], the maintenance of stability for a long term is an importance issue [27,28,29]. Like LDH, ADH easily loses its activity under freeze–thaw stress [29,30].

In this study, we hypothesized that the protective capability of IBPs on proteins against freeze–thaw damage is proportional to their IRI activity. To test the hypothesis, we used ADH as a model freeze-labile protein. The protection of ADH against freeze–thaw damage in the absence and presence of IBPs was assessed by measuring its residual activity during storage. Here, we not only confirmed that the IRI activity of IBPs was essential for protecting ADH from the freeze–thaw damage, but also demonstrated that IRI activity was correlated with IBP’s protective effect on ADH.

## 2. Results and Discussion

### 2.1. IBPs Show Resistance to the Repeated Freeze–Thaw Cycle

**Figure 1:**
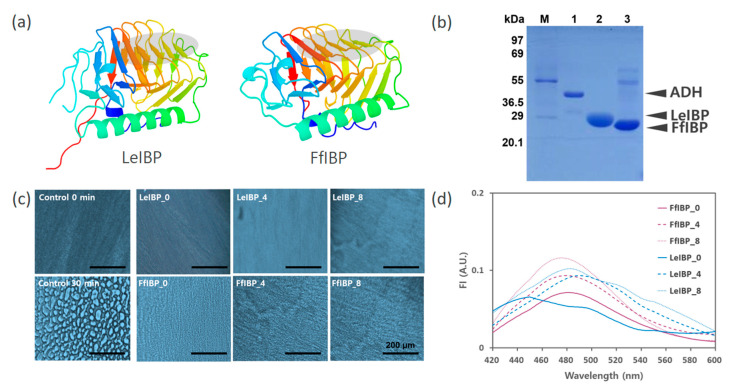
Structure, sodium dodecyl sulfate polyacrylamide gel electrophoresis (SDS-PAGE), and ice recrystallization inhibition (IRI) and stability after repeated freeze–thaw cycle of two ice-binding proteins (IBPs) used in this study. (**a**) Cartoon diagram of two IBPs: LeIBP (PDB ID 3UYU) from Arctic yeast *Glaciozyma* sp. and FfIBP (PDB ID 4NU2) from Antarctic bacterium *Flavobacterium frigoris*. Ice-binding site is displayed as a gray oval. (**b**) SDS-PAGE (12%) of proteins used in this study. M: molecular weight makers; Lane 1, alcohol dehydrogenase from baker’s yeast; Lane 2, purified LeIBP; Lane 3, purified FfIBP. (**c**) Ice recrystallization inhibition assay of Le- and FfIBP at 0.1 mg mL^−1^ after four and eight freeze–thaw cycles. Control was 10 mM sodium phosphate buffer (pH 7.5). Scale bar = 200 μm. (**d**) Fluorescence emission spectra of ANS in the two IBPs subjected to four and eight freeze–thaw cycles. The concentrations of ANS and IBPs were 100 μM and 3.85 μM, respectively.

To investigate the protective effect of IBPs on freeze-labile ADH, we selected two IBPs of similar tertiary structure but of different IRI activity (Figure 1a) [18,19]. The recombinant LeIBP was about 25-fold active in IRI compared with FfIBP. As shown in Figure 1b, both IBPs were purified to near homogeneity, except for the minor contamination in the FfIBP sample, and concentrated up to 8 mg mL^−1^. First we examined their stability over the repeated freeze–thaw cycles. All the freeze–thaw processes in this study were performed in a household freezer with no freezing rate controller, which may maximize the freeze–thaw damage to proteins by relaxing good lab practice. The freeze–thaw damage to IBPs was examined using the IRI activity assay and the structural perturbation probed with 1-anilinonaphthalene-8-sulfonate (ANS), a hydrophobic fluorescent probe [31,32]. The IRI activity (Figure 1c) using a splat cooling assay method showed that none of freeze–thawed IBP samples (0.1 mg mL^−1^) were affected by the freeze–thaw cycles. The fourth and eighth repeatedly freeze–thawed Le- and FfIBP exhibited no discernible difference to unfrozen proteins in IRI. This result is not surprising because for a decade of studying IBPs, the authors have noticed no diminution of their activity over the several freeze–thaw cycles during TH measurement on a cold stage. The ANS dye binding to hydrophobic regions of a protein has been widely used to follow unfolding of a protein by a variety of stresses [32,33,34,35]. Freeze–thawed IBPs were also mixed with ANS and its emission spectra were obtained (Figure 1d). If freeze–thaw caused unfolding or perturbation in the tertiary structure of IBPs, more hydrophobic regions were exposed in IBPs that would lead to the increase in the fluorescence emission spectra of ANS. There were a slight shift and increase but no marked increase in fluorescence spectra in the freeze–thawed samples. These data were in accordance with the recent finding with a beetle AFP [10]. Very similar to this work, they acquired almost identical circular dichroism spectra of DcAFP before and after four freeze–thaw cycles. In all, we believe that both Le- and FfIBP maintain their structural and functional integrity at least up to eight repeated freeze–thaw cycles.

### 2.2. ADH Is Susceptible to the Repeated Freeze–Thaw Cycles

**Figure 2:**
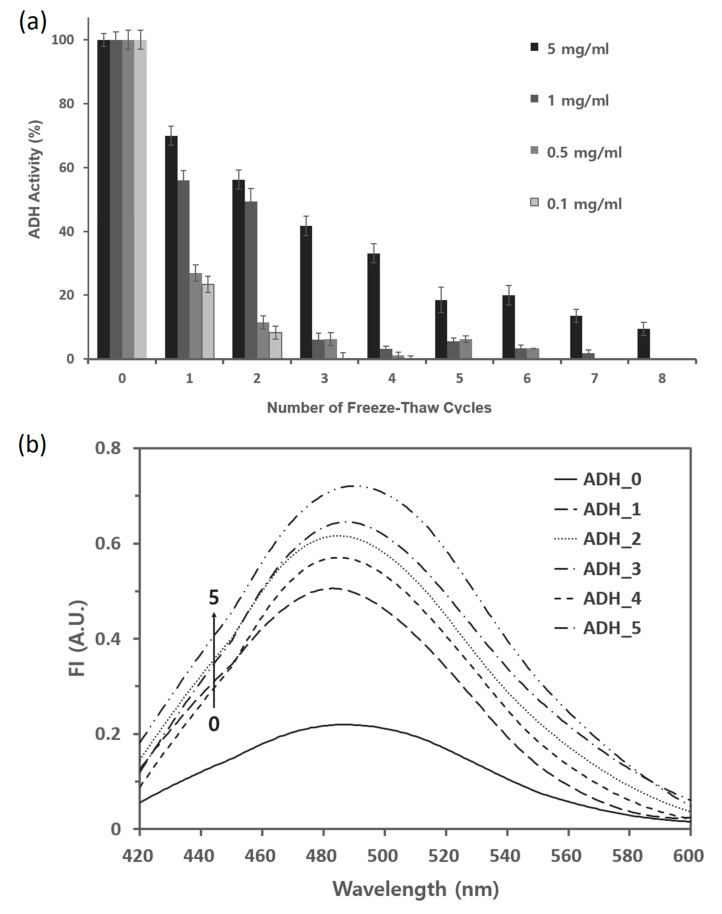
Activity and fluorescence emission spectra of alcohol dehydrogenase (ADH) after repeated freeze–thaw cycles in the absence of ice-binding proteins (IBPs). (**a**) The activity of ADH was recorded by measuring the increase in absorbance at 340 nm due to the reduction of NAD^+^ to nicotinamide adenine dinucleotide (NADH) in the 50 mM sodium phosphate buffer (pH 8.8). The ADH activity was expressed as a percentage of the activity of the unfrozen control ADH sample. (**b**) Fluorescence emission spectra of ADH in the buffer subjected to the repeated freeze–thaw cycles were obtained using 1-anilinonaphthalene-8-sulfonate (ANS) as a hydrophobic fluorescent probe. The concentrations of ANS and ADH were 100 μM and 6.8 μM, respectively.

The effect of ADH concentration during freeze–thaw cycles was first investigated. The freeze–thaw cycle was the same as in the case of IBPs. The dependence of freeze-induced inactivation on ADH concentration is shown in Figure 2a. The loss of activity was drastically prominent at lower concentrations, but gradually decreased at higher concentrations. This phenomenon agrees with previous findings [30,36,37]. On the contrary, both thermal [38] and urea [39] inactivation of yeast ADH was independent of enzyme concentrations, indicating that dissociation of tetrameric enzyme has no effect on the rates of inaction. However, the concentration dependence of ADH activity during freeze–thaw cycles could result from the aggregated proteins on the surface of ice, which lead to the inactivation or denaturation [13,40]. Based on Figure 2a, we chose 5 mg mL^−1^ of ADH for the rest of the experiment, since this concentration was sensitive to freeze–thaw and high enough to minimize errors for the activity assay and to perform the fluorescence experiment. Fluorescence emission spectra of ADH after repeated freeze–thaw cycles are shown in Figure 2b. The fluorescence intensity continuously increased with increasing freeze–thaw cycles. In addition, the slight red-shift of fluorescence maximum was observed as in the urea and thermal denaturation of ADH [38,39]. The red-shift implied that the Tyr and Trp residues in ADH were exposed during denaturation. The current fluorescence data may indicate that the denaturation mechanism induced by the freeze–thaw cycle is almost identical to those caused by other stresses.

### 2.3. Activity of ADH Is Rescued Proportional to the IRI Property of IBPs

**Figure 3:**
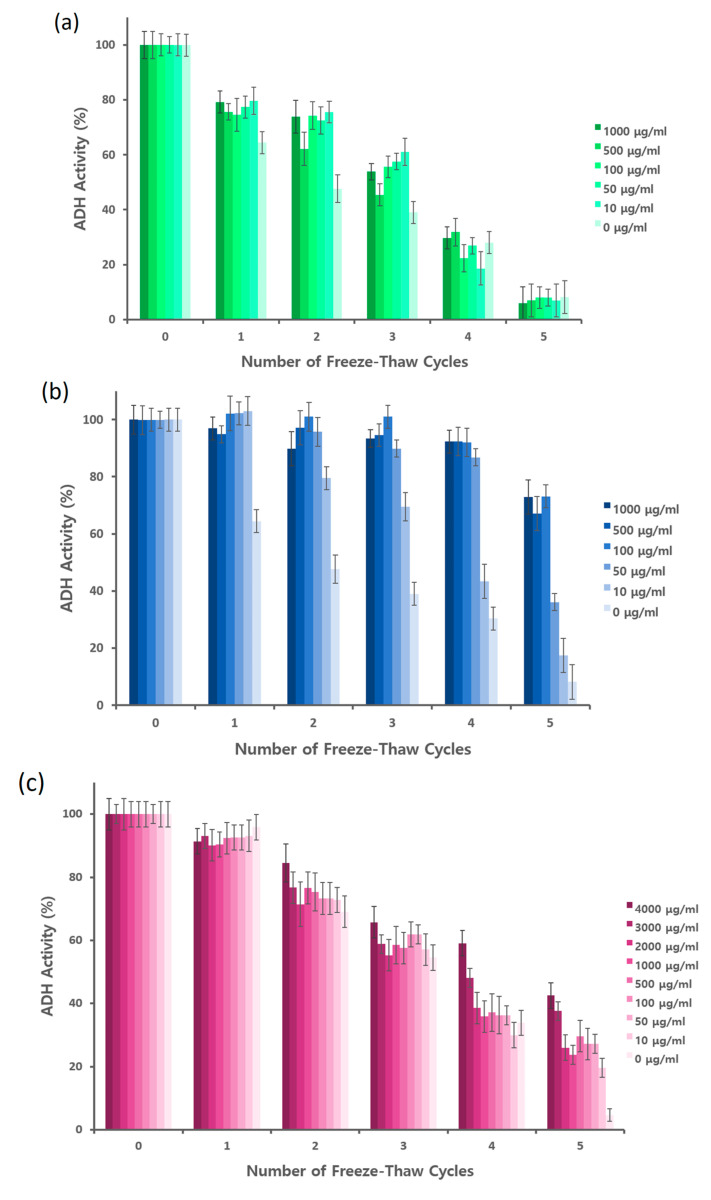
Activity of alcohol dehydrogenase (ADH) after repeated freeze–thaw cycles in the presence of varying concentrations of bovine serum albumin (**a**), Arctic yeast *Glaciozyma* sp. IBP (LeIBP) (**b**), or Antarctic bacterium *Flavobacterium frigoris* IBP (FfIBP) (**c**). The concentration of each protein additive is displayed using a color gradient. The color of the higher concentration is displayed darker than the lower one. The activity of ADH was recorded by measuring the increase in absorbance at 340 nm due to the reduction of NAD^+^ to NADH, and the ADH activity was expressed as a percentage of the activity of the unfrozen control ADH sample.

The protective effect of IBPs on ADH subjected to freeze–thaw cycles was assessed by measuring the remaining activity. Five mg mL^−1^ ADH solution in the 10 mM sodium phosphate buffer (pH 7.5) was mixed with varying concentration of bovine serum albumin (BSA) as a control, LeIBP, or FfIBP. As shown in Figure 3a, BSA did not protect ADH from freeze–thaw stress. This observation was rather different from the case of lactate dehydrogenase (LDH) [10], because 5 mg mL^−1^ BSA could rescue 50% of the original activity of LDH after six freeze–thaw cycles. In the ADH case, we used up to 1 mg mL^−1^ BSA to directly compare the protective effect with IBPs. Hence the direct comparison is not appropriate. Expectedly, the addition of LeIBP drastically improved the activity of ADH (Figure 3b). As the concentration of LeIBP increased, the remaining activity of ADH was also increased. After five cycles of freeze–thaw, about 70% of the ADH activity remained in the LeIBP concentration of 100 ~ 1000 μg mL^−1^, while only 36% and 17% were at 50 and 10 μg mL^−1^, respectively. From this data the minimal concentration for effective protection of ADH is 100 μg mL^−1^. This concentration corresponds to the minimal effective concentration for the cryopreservation of animal cells [41] and microalgae [42]. Interestingly, the protective effect of FfIBP was much lower than that of LeIBP (Figure 3c). FfIBP treatment showed relatively higher remaining activity compared to BSA treatment after the first and fifth freeze–thaw but did not show any significant improvement during the second to fourth freeze–thaw cycles. FfIBP exhibited protective effect at higher concentrations (>1 mg mL^−1^). This result may strongly stress the importance of IRI activity in protection against freeze–thaw damage. FfIBP has higher TH activity, but is about 25-fold lower in IRI activity compared to LeIBP [19]. From a few reports on cryopreservation [20,42], LeIBP generated more beneficial results than FfIBP even though it had lower TH activity. This is generally attributed to the phenomenon whereby the post-thaw viability of cryopreserved cells can decrease due to the needle-like ice formation at higher concentrations of certain IBPs [20,21,23,43,44]. Noticeably, hyperactive FfIBP shows a burst of ice crystal when the TH gap is exceeded, which may be detrimental to frozen proteins or cells. Contrary to our observation, DcAFP, having hyperactivity, showed a protective effect on LDH at 85 μg mL^−1^ and rescued 90% LDH activity even after the sixth freeze–thaw cycle. The possible interaction of DcAFP with tetrameric LDH during freeze–thaw using molecular dynamics simulation by which DcAFP further protects LDH activity has been proposed. However, IBPs used in this study may not interact with ADH, since only a high concentration of FfIBP showed a protective effect to a certain degree. Recently, Mitchell et al. [13] reported the importance of IRI activity for the protection of proteins against freeze stress. AFP-inspired IRI-active polymers poly (vinyl alcohol) and polyampholyte with poly-(ethylene glycol) were successfully used for the cryopreservation of insulin, Taq DNA polymerase, and IgG antibody. This indicated that the IRI-active polymers may also protect proteins against freeze stress. Currently, the possible mechanism of protection against freeze–thaw stress by IBPs is still not known clearly. Rodriguez et al. [10] proposed the interaction of DcAFP with LDH and water–ice interfaces, while Mitchell et al. [13] suggested the prevention of irreversible aggregation due to ice formation. To clarify the mechanism, further studies on interactions between an IBP and proteins in ice and ice during the freezing and thawing process need to be investigated.

In this study, we investigated the potential protective effect of IBPs on ADH against freeze–thaw stress. To elucidate the possible mechanism and the different behavior of two IBPs, further microscopic investigations such as interactions of IBPs with a protein and ice in the frozen protein solution will be needed in the near future. We also believe this work will encourage the development of IBP-inspired IRI-active molecules as cryoprotective agents for proteins [45,46,47,48].

## 3. Materials and Methods

### 3.1. Chemicals and Proteins

All chemical reagents used in this study, including β-NAD, were obtained from Sigma Chemical Co (St. Louis, MO, USA). Alcohol dehydrogenase (ADH) from *Saccharomyces cerevisiae* was also purchased from Sigma Chemical (St. Louis, MO, USA). Bovine serum albumin (BSA) was purchased from Biogen (Cambridge, MA, USA). The prescribed amount of ADH and BSA was dissolved in 10 mM sodium phosphate buffer (pH 7.5) and incubated for 1 h at room temperature just prior to use. Two ice-binding proteins (IBPs) used in this study were produced as described elsewhere [18,19]. In brief, firstly, the overexpression of LeIBP from the *Pichia pastoris* X33 cell was induced by 5 mL of methanol per day for 6 days. The secreted LeIBP in the culture medium was purified by the anion exchange Q-Sepharose FF column (GE Healthcare, Little Chalfont, UK) and subsequent Superdex 200 size-exclusion column (GE Healthcare, Little Chalfont, UK). Secondly, the FfIBP cloned in pCold1 vector was overexpressed *Escherichia coli* BL21 strain (Invitrogen, Carlsbad, CA, USA) by the addition of 1 mM isopropyl β-D-1-thiogalactopyranoside. Immediately, the temperature was shifted to and left at 16 °C overnight to increase the soluble expression of FfIBP. The FfIBP was purified using an Ni-NTA affinity chromatography. The purified LeIBP and FfIBP were dialyzed against 10 mM sodium phosphate buffer (pH 7.5) and concentrated using a Centricon 10 K filter (Milipore, MA, USA) up to 8 mg mL^−1^. The purity of ADH and the purified two IBPs was confirmed by sodium dodecyl sulfate polyacrylamide gel electrophoresis (SDS-PAGE) [49], and their concentration was determined by Bradford method [50].

### 3.2. Ice Recrystallization Inhibition Assay

A splat cooling assay introduced by Dr. Charles Knight was used to assess the ice recrystallization inhibition property of two IBPs [31]. The concentrated IBP solutions were diluted in 10 mM sodium phosphate buffer (pH 7.5). From a height of 2 m, a 10 μL droplet containing 0.1 mg mL^−1^ of IBP was dropped onto a polished aluminum block pre-cooled with dry ice. Then the ice wafer was instantly formed on the aluminum block. The wafer was placed in a Linkam LTS120 cold stage (Linkam Scientific Instruments Ltd., Surrey, UK) maintained at −6 °C. The ice wafer annealed for 30 min and its images were captured at 0 and 30 min by the Linkam Imaging Station. The buffer was used as a control.

### 3.3. Freeze–Thaw of ADH and Its Activity Assay

The stock solution (10 mg mL^−1^) of ADH was prepared in 10 mM sodium phosphate buffer (pH 7.5). To find out the appropriate enzyme concentration for the assay after the freeze–thaw experiment, we prepared 0.1, 0.5, 1, and 5 mg mL^−1^ ADH solutions. One mL of the enzyme solution of each concentration was dispensed into 1.5 mL microcentrifuge tubes and frozen at −20 °C by placing them directly in a freezer. The enzyme samples were kept frozen for 24 h to ensure the complete freezing, and then were thawed at room temperature for 2 h. The thawed sample was used to check the activity. This freeze–thaw was repeated eight times. To evaluate the protective effect of IBPs on the freeze–thaw damage of ADH, 5 mg mL^−1^ ADH was mixed with BSA, LeIBP, or FfIBP. The final concentration of BSA or LeIBP added was 10, 50, 100, 500, and 1000 μg mL^−1^, while that of FfIBP was 10, 50, 100, 500, 1000, 200, 3000, 4000 μg mL^−1^. Freeze–thaw was performed as described earlier. One hundred μL freeze–thawed ADH solution from each cycle was used for the ADH activity assay. Freeze–thawed samples with no additives were used for both the activity assay and ANS fluorescence measurement. All experiments were carried out in triplicate.

The activity of ADH was examined at 25 °C by observing the increase of absorbance at 340 nm as a result of the reduction of NAD^+^ to nicotinamide adenine dinucleotide (NADH) according to the manufacturer’s protocol. The reaction mixture contained 50 mM sodium pyrophosphate buffer (pH 8.8) 450 μL, 95% (*v*/*v*) ethanol 40 μL, and 15 mM β-NAD 500 μL. The reaction was initiated by adding 10 μL of enzyme sample containing 50 μg mL^−1^ ADH and monitored using BioDrop Duo spectrophotometer (Cambridge, UK). Initially the specific enzyme activity (μmol min^−1^ mg^−1^) was calculated from the slope and the residual activity of freeze–thawed samples was expressed as a percentage of the activity of the unfrozen control ADH sample.

### 3.4. ANS Fluorescence Measurement

To monitor the freeze–thaw-induced perturbations of protein tertiary structure 1-anilinonaphthalene-8-sulfonate (ANS) was used as a fluorescence probe [32]. Fluorescence spectra was recorded using a JASCO fp-6300 spectrophotometer (Tokyo, Japan) with 1 cm path-length cuvette. The excitation wavelength was 350 nm and the emission wavelengths was 420–600 nm. The ANS stock solution was freshly prepared in water to the concentration of 0.03 mg·mL^−1^. The 5 mg mL^−1^ ADH solution in the 10 mM sodium phosphate buffer underwent repeated freeze–thaw cycles as described above. After each freeze–thaw cycle, 50 μL ADH solution was mixed with 100 μL ANS stock solution and 850 μL buffer, and equilibrated for 10 min at room temperature before measurement. The IBP fluorescence spectra were measured to be the same as the those of ADH. The IBP concentration used for the freeze–thaw cycle was 0.1 mg·mL^−1^ for both IBPs. All fluorescence spectra were corrected by subtracting a buffer spectrum as a blank.

## 4. Conclusions

In this study, we examined the hypothesis that the protective capability of IBPs on proteins against freeze–thaw damage is proportional to their IRI activity using ADH as a freeze-labile protein. IBPs with high (LeIBP) and low (FfIBP) IRI activity were used. As expected, IBPs alone showed no sign of cold-denaturation during repeated freeze–thaw cycles. But not surprisingly, freeze-labile ADH alone appeared to be unfolded during the process, demonstrated by the residual enzyme activity and fluorescence emission spectra probed by ANS. However, the addition of IBPs increased the residual activity of ADH after repeated freeze–thaw cycles: 80% residual activity at a minimal concentration of 0.1 mg mL^−1^ of high IRI-active LeIBP, and 50% activity at 4 mg·mL^−1^ of low active FfIBP. The protection of ADH from freeze–thaw damage was distinct in high active LeIBP compared to FfIBP. To our knowledge, this is the first study showing that the protection of proteins from freeze–thaw stress by IBPs is proportional to their IRI activity. Currently the mechanism of protection by IBP is still unclear. Further studies on protection mechanism may provide clues for the rational development of non-toxic and biocompatible cryoprotective agents for protein therapeutics and industrial enzymes.

## Abbreviations

ADH, alcohol dehydrogenase; AFP, antifreeze protein; ANS, 1-anilinonaphthalene-8-sulfonate; BSA, bovine serum albumin; DcAFP, *Dendroides canadensis* AFP; FfIBP, IBP from *Flavobacterium frigoris*; IBP, ice-binding protein; IgG, immunoglobulin G; IR, ice recrystallization; IRI, IR inhibition; LDH, lactate dehydrogenase; LeIBP, IBP from *Glaciozyma* sp.; NADH, nicotinamide adenine dinucleotide; NTA, nitrilotriacetic acid; PDB, protein data bank; SDS-PAGE, sodium dodecyl sulfate polyacrylamide gel electrophoresis; Taq, *Thermus aquaticus*; TH, thermal hysteresis
